# Rectal hemangiopericytoma in a 37-year-old woman: a case report and review of the literature

**DOI:** 10.1186/1752-1947-5-352

**Published:** 2011-08-05

**Authors:** Li Lu, Long Jiang Zhang, Chang Sheng Zhou, Guang Ming Lu

**Affiliations:** 1 Department of Medical Imaging, Jinling Hospital, Nanjing, Jiangsu 210002, China

## Abstract

**Introduction:**

Hemangiopericytoma is an uncommon perivascular tumor. Rectal Hemangiopericytomas are extremely rare. To the best of our knowledge, only two cases have been reported in the literature.

**Case presentation:**

We report the case of a 37-year-old Asian woman with an Hemangiopericytoma rising from the anterior wall of her rectum. Abdominopelvic computed tomography showed a 7.4 cm solid mass between her uterus and her rectum. Heterogeneous gradual enhancement after intravenous injection of contrast material was noted with several tortuous vessels around her tumor. Intra-operative findings indicated a capsule and well-circumscribed solid tumor connecting with the anterior wall of her rectum by a small pedicle. With immunohistochemical stains, her tumor cells reacted positive for Bcl-2, CD34, and ki67 and negative for CD10, CD117, S100, and Desmin. Follow-up computed tomography scans have shown no tumor recurrence or metastasis signs.

**Conclusions:**

Rectal Hemangiopericytoma is a rare tumor with non-specific imaging findings. Hemangiopericytomas should be included in the differential list when a massive tumor with heterogeneously gradual enhancement in the regions of the rectum is encountered.

## Introduction

Hemangiopericytoma (HPC), an uncommon perivascular tumor, accounts for 1% of primary vascular tumors and occurs most frequently in the extremities, pelvis, head and neck, and meninges [1]. This tumor is generally rare in the gastrointestinal tract. Rectal HPC is extremely rare; to the best of our knowledge, only two cases have been reported in the literature in English [2]. It has been reported that some HPCs rising from the sacrum involved merely the retrorectal space [3]. Few reports on radiological findings of rectal HPCs have been published. Here, we report the clinical, ultrasonongraphy, and dynamic contrast-enhanced computed tomography (CT) findings of an HPC rising from the rectal anterior wall of a 37-year-old woman.

## Case presentation

**Figure 1:**
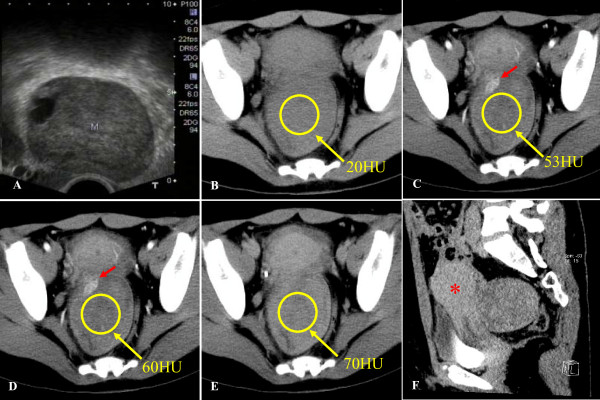
**(A) Intra-vaginal ultrasonography of a pelvic mass**. Intra-vaginal ultrasonography showed a 6.0 × 7.6 × 6.0 cm solid mass (M) between the uterus and the rectum. Dynamic contrast-enhanced computed tomography (CT) of the pelvis was used. (B) A non-enhanced CT scan showed the mass between the uterus and the rectum with nearly homogeneous density with a CT number of 20 Hounsfield units (HU). (C) Arterial phase, (D) venous phase, and (E) delayed phase contrast-enhanced CT showed that CT numbers of regions of interest within the mass (oval) gradually enhanced from 53 to 60 to 70 HU, respectively. A marked contrast-enhanced structure (red arrow) corresponding to tortuous vessels was shown with CT values of 140.0 HU in the arterial phase (C) and 111.6 HU in the venous phase (D). (F) Sagittal multi-planar reformation showed the mass (arrow) anteriorly growing and deforming the uterus (*).

**Figure 2:**
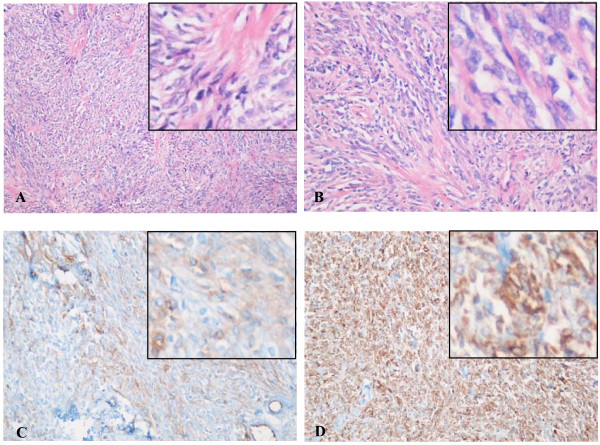
**Histopathological images**. (A) Collagen denaturation can be seen in a partial mesenchyme. Branch-like blood vessels are visible (hematoxylin and eosin [H-E] stain, original magnification ×100). (B) Spindle and oval tumor cells and stromal sinusoid can be observed (H-E stain, original magnification ×200). (C) In CD34 immunohistochemical stains, tumor cells reacted positive for CD34. (D) In Bcl-2 immunohistochemical stains, tumor cells reacted strongly positive for Bcl-2.

A 37-year-old Asian woman was referred to our hospital because of lower abdominal pain that began four months earlier. A vaginal palpation revealed a hard, adhering, and painless mass. Another physical examination revealed no abnormalities. The results of laboratory tests, including complete blood count, serum electrolytes, creatinine, and urea, were normal. Our patient underwent an intra-vaginal ultrasonography (US) examination, which revealed a 6.0 × 7.6 × 6.0 cm solid mass between her uterus and rectum (Figure 1A). An abdominopelvic CT scan showed a 7.4 cm nodular solid mass between her uterus and rectum and an intense heterogeneously gradual enhancement after intravenous injection of iodinated contrast material. CT numbers of the mass ranged from 20 Hounsfield units (HU) in unenhanced CT to 70 HU in the delayed phase (Figure 1B-E). Tortuously enhanced vessels around her tumor were also noted (Figure 1C, D). The mass encroached into the posterior part of her uterus prominently (Figure 1F) but without involving adjacent organs. No lymphoadenopathy was found. Subserosal uterine fibroid was suspected at CT. Our patient underwent tumor resection after a comprehensive evaluation of clinical and imaging findings. Intra-operative findings indicated a capsule and well-circumscribed solid tumor connecting with the anterior wall of her rectum by a small pedicle. The gross specimen showed a well-encapsulated mass that was 10.0 × 8.0 × 5.0 cm in size. The external surface was pink and whitish. Microscopically, the specimen showed the features of a mesenchymal tumor with spindle and oval cells (Figure 2A). Branch-like blood vessels were visible within the tumors (Figure 2B). With immunohistochemical stains, tumor cells reacted positive for CD34 (Figure 2C), Bcl-2 (Figure 2D), and ki67 and negative for CD10, CD117, S100, and Desmin. The tumor had low malignant potential activity. Follow-up pelvis US and CT examinations revealed no tumor recurrence or metastasis signs six months after surgery.

## Discussion

HPC was first described in 1942, by Stout and Murray [4], and has been further understood since the development of electron microscopy, immunohistochemistry, and cytogenetics in the 1970s. HPC is classified as a soft-tissue vascular tumor arising from pericytes, which are contractile cells surrounding the capillaries and post-capillary venules [5]. Consequently, HPC may occur anywhere capillaries are found. Rectal HPC is very rare; to the best of our knowledge, only two cases rising from the rectum have been described in the literature [2]. The tumor can present in patients of any age but does so predominantly in the fourth and fifth decades and has a male-to-female ratio of 1.8.

HPCs have some characteristic clinical features. One of these features is the rate of recurrence, which is as high as 52% of cases [6] (mostly in the lungs, liver, and regional lymph nodes) and which necessitates long-term follow-up after resection of the primary tumor. Other interesting features are the various para-neoplastic symptoms, including hypoglycemia [7] and hypertension [8], which accompany this neoplasm because the tumor can secrete insulin-like substances and hyper-utilize glucose. A review of the literature revealed that the size of a tumor causing hypoglycemic symptoms ranged from 12 to 27 cm. In our patient, the size of the primary tumor was 10.0 × 8.0 × 5.0 cm.

The radiographic features of rectal HPCs are non-specific. A large HPC usually has a marked mass effect with necrosis and cystic changes. Calcification is rare. Intense heterogeneous gradual enhancement can be observed after intravenous injection of contrast material with several tortuous enhanced vessels around the tumor, which indicate the vascular origin of the tumor. The uncertainty of the rectal origin reflects the large exophytic nature of the tumor and its relatively small pedicle [9]. Magnetic resonance imaging (MRI) is usually chosen as the method for detecting the organ of origin of a pelvic mass. However, MRI was not performed in our patient. On MRI, HPC typically shows an intermediate signal intensity on T1-weighted images and hyper-intense serpentine channels on gadolinium-enhanced images. MRI shows a characteristic sign-"flow void phenomena"-that often emerges from hyper-vascular tumors. Lipomatous HPCs are benign variants of HPCs [10].

Rectal HPCs need to be differentiated from three types of tumors: uterine myomas, exogenous gastrointestinal stromal tumors (GISTs) of the rectum, and retroperitoneal tumors. On MRI, non-degenerating uterine myomas show entirely or predominantly low signal intensity on T2-weighted images, and it displays differentiation between uterine myomas and HPCs because HPCs appear as high signal intensity on T2-weighted images. But degenerated uterine myomas may have varied appearances on T2-weighted and contrast-enhanced images according to the hyaline or myxoid degeneration, degree of interstitial edema, cystic degeneration, necrosis, fibrosis, calcification, hemorrhage, carneous degeneration, and fat.

Small tumors typically appear as homogeneous soft-tissue masses with moderate contrast enhancement, whereas large tumors often appear to have a heterogeneous density or signal intensity because of ulceration, necrosis, or cavitation. Thus, precise differential diagnosis is very difficult, but GISTs rarely cause lymph node metastasis; if extensive lymph node metastases are found, other diseases should be considered [11].

It is very difficult to differentiate retroperitoneal tumors, such as leiomyosarcoma, liposarcoma, neurogenic tumors, and malignant fibrous histiocytoma (MFH), on the basis of imaging findings. Of these tumors, liposarcoma is one of the most common primary neoplasms in the retropenitoneum. The lipoma-like component may lead to a diagnosis of liposarcoma, although abdominal tumors with fat are not always liposarcomas [12]. Neurogenic tumors often occur in lateral walls of the pelvis with moderate or marked enhancement. MFH and leiomyosarcoma have non-specific imaging findings, which do not facilitate a definitive diagnosis; however, a low-signal-intensity septum of the tumor in T2-weighted images may indicate a diagnosis of MFH [13].

Tumor resection is the mainstay method of treatment of HPCs. Post-operative adjuvant radiotherapy should be offered to all patients, regardless of the degree of resection. The optimal management of local recurrence is indicated by the size of the recurrence and the overall systemic disease burden present at the time of recurrence. Post-operative radiation therapy does not confer any significant protection against the development of distant metastases. For this reason, long-term clinical and radiographic follow-up of these patients is imperative given that recurrence or metastasis or both often take several years to develop.

## Conclusions

Rectal HPC is a rare tumor with non-specific imaging findings. HPCs should be included in the differential list when a massive tumor with heterogeneously gradual enhancement in the regions of the rectum is encountered.

## Abbreviations

CT: computed tomography; GIST: gastrointestinal stromal tumor; HPC: hemangiopericytoma; HU: Hounsfield units; MFH: malignant fibrous histiocytoma; MRI: magnetic resonance imaging; US: ultrasonography.

## Consent

Written informed consent was obtained from the patient for publication of this case report and any accompanying images. A copy of the written consent is available for review by the Editor-in-Chief of this journal.

## Competing interests

The authors declare that they have no competing interests.

## Authors' contributions

LL gathered the data, performed the literature review, and edited the manuscript. LJZ and CSZ participated in the acquisition and analysis of the literature data and helped to draft the manuscript. GML revised the final manuscript. All authors read and approved the final manuscript.
